# Vegan vs. omnivore diets paradox: A whole-metagenomic approach for defining metabolic networks during the race in ultra-marathoners- a before and after study design

**DOI:** 10.1371/journal.pone.0255952

**Published:** 2021-09-23

**Authors:** Aslı Devrim-Lanpir, Havvanur Yoldaş İlktaç, Katharina Wirnitzer, Lee Hill, Thomas Rosemann, Beat Knechtle

**Affiliations:** 1 Department of Nutrition and Dietetics, Faculty of Health Science, Istanbul Medeniyet University, Istanbul, Turkey; 2 Department of Subject Didactics and Educational Research and Development, University College of Teacher Education Tyrol, Innsbruck, Austria; 3 Department of Sport Science, Leopold-Franzens University of Innsbruck, Innsbruck, Austria; 4 Research Centre Medical Humanities, University of Innsbruck, Innsbruck, Austria; 5 Life and Health Science Cluster Tyrol, Subcluster Health/Medicine/Psychology, Tyrolean University Conference, Innsbruck, Austria; 6 Division of Gastroenterology & Nutrition, Department of Pediatrics, McMaster University, Hamilton, Canada; 7 Institute of Primary Care, University of Zurich, Zurich, Switzerland; 8 Medbase St. Gallen Am Vadianplatz, St. Gallen, Switzerland; Oregon State University, UNITED STATES

## Abstract

**Background:**

The effect of vegan diets on metabolic processes in the body is still controversial in ultra endurance athletes. The study aims to determine gut microbiome adaptation to extreme exercise according to vegan or omnivore diet consumed in ultra-marathoners. We also seek to evaluate long-term vegan diets’ effects on redox homeostasis, and muscle fatigue, and assess energy availability.

**Methods:**

Seventy participants will be assigned to the study, including 35 vegan ultra-marathoners and 35 omnivores competing in the Sri-Chinmoy ultra marathon race. Research data will be collected from the participants at four steps (three visits to the research laboratory and the race day) throughout the study. At the first visit (seven days before the race), fecal samples, and anthropometric measurements will be collected. Body composition will be measured using DXA. Participants will be informed about keeping detailed food records and will be asked to record their diet data and activity logs during the entire study period. At second visit, maximum oxygen consumption will be measured on treadmill. On race day, blood samples will be collected immediately before, and 0. min, 2 hours, and 24 hours after the race. Body weight will be measured before and after the race. The blood and fecal samples will be stored at -80 C until analysis. Plasma malondialdehyde, reactive oxygen metabolites, total antioxidant capacity, Heatshockprotein-70, and serum Orosomucoid-1 will be analyzed in blood samples. Fecal samples will be analyzed with shotgun metagenomic analysis and interpreted using bioinformatics pipeline (HumanN2). Statistical tests will be analyzed using SPSS version 23.0 and R Software.

**Discussion:**

Study findings will determine the effects of the vegan diet on sports performance, revealing the multiple interactions between host and gut microbiome at the whole metagenomic level. Additionally, results will show the possible adaptation throughout the race by analyzing blood and fecal samples. Furthermore, by assessing energy availability and determining host-metabolite crosstalk for ultra-endurance athletes, possible nutritional deficiencies can be identified. Thus, advanced nutritional strategies can be developed based on metabolic needs.

**Trial registration:**

Current controlled trials, ISRCTN registry 69541705. Registered on 8 December 2019.

## Introduction

With several elite athletes adopting veganism, a vegan diet has become more popular in the athletic population [1]. The vegan diet, which eliminates all animal products from an individual’s diet, promotes health benefits that include a lower risk of cardiovascular disease, diabetes, obesity, hypertension, and cancer [2–4]. However, veganism has raised concerns about possible nutrient deficiencies, mainly protein, w-3, vitamin B12, zinc, and calcium [4, 5]. Although the popularity of the vegan diet has increased among athletes, its effects on health and performance are still contentious. Additionally, there is a lack of research into the vegan dietary pattern in the athletic population [6].

Exhaustive exercise and inadequate recovery in the ultra-endurance athletes have been associated with the excessive production of lipid peroxidation and ROS, which alters body hormesis and causes both cellular and tissue damage [7, 8]. The excessive release of stress factors causes an increase of lipopolysaccharides (LPS) translocation outside of the gut, resulting in exercise-induced endotoxemia, thus increasing permeability [9]. While lipid peroxidation and ROS can be determined in the formation of metabolic products such as malondialdehyde (MDA) [10], reactive oxygen metabolites (d-ROMs) [11], the body’s endogenous antioxidant capacity can be evaluated by measuring plasma total antioxidant capacity (TAC) [12], a whole measurement of both enzymatic and non-enzymatic antioxidants. Previous studies have reported that consuming fruits and vegetables rich in antioxidants instead of taking antioxidant supplementation is suggested as the best approach to maintaining the body’s oxidative balance [13–15]. A vegan diet, rich in both antioxidants and flavonoids, which are naturally occurring anti-inflammatory factors [1], could be an effective strategy to regulate the microbiome, reduce muscle fatigue and improve performance in ultra-endurance athletes.

Muscle fatigue is a common phenomenon caused by an alteration in metabolic conditions that restricts life and has detrimental effects on exercise performance [16]. Detection of muscle fatigue-related biomarkers, thus developing potential strategies to reduce muscle fatigue, is crucial for managing the pathologic conditions and enhancing muscle endurance in athletes, particularly during exhaustive exercise [17]. Immunologic biomarkers such as heat shock proteins (HSPs) [18] and orosomucoid (ORM) [19] are classified as a novel strategy to modulate muscle fatigue and immuno-modulating activity. In addition, Wan et al. [20] has claimed that foods rich in vitamins, minerals, flavonoids, and w-3 fatty acids are promising dietary factors that may reduce muscle fatigue in athletic populations. More research is needed to determine the effects of vegan and omnivorous diets on exercise-induced muscle fatigue and immunological conditions in athletes.

As long-term, low-grade energy and both macro- and micronutrient deficiencies are common in ultra-endurance athletes, they are at high risk for the occurrence of relative energy deficiency syndrome (RED-S) [21], including a decrease in bone mineral density and endocrine dysfunction, and reduced musculoskeletal function [22]. Previous literature has emphasized that vegans consume less energy and macro- and micronutrients than omnivores [3]. Whether vegan diets provide sufficient energy availability and preserve bone mineral density is a particular concern for vegan athletes, especially in ultra-endurance races.

Studies investigating the interaction between the gut microbiome and exercise performance in athletes have gradually increased over the past decades by searching the primary role of the gut microbiome on energy metabolism, immunomodulation, inflammatory response, and oxidative stress regulation [23]. Prior investigations have reported that exercise alone may contribute to alterations in the gut microbiome composition [24, 25]. However, little is known about the interactions of microbial communities with exercise-related metabolic systems and communication pathways for exercise-related modification. Scheiman et al. [26] emphasized that microbiome modulation by detecting its performance-facilitating organism may be a critical exercise performance component. Metagenomic analyses allow direct comparison of the metabolism in the intestinal microbiota with the host’s metabolic results and, integration of metabolomics and microbiome analysis could provide us to identify the microbial influence on host metabolism through bioactive metabolites [27–29]. Although a vegan diet appears to be beneficial for the gut microbiome by consuming foods that modulate/ regulate the microbiome [30], further work should focus on the efficacy of a vegan diet on host-microbiome interactions. To our knowledge, no study determines the vegan vs. omnivore diets’ effects on gut metagenomics analyzed using a whole-metagenomic approach.

### Trial objectives

The aims of the study are:

1. To investigate the effects of long-term vegan and omnivorous diet on gut metagenomics in ultra-marathoners,
2. To evaluate the adaptation of the gut microbiome after extreme endurance exercise according to a vegan or omnivorous diet in ultra marathoners,
3. To compare the differences between vegan and omnivorous ultra-marathoners.
4. To investigate the effects of vegan and omnivorous diets on exercise-induced oxidant/antioxidant capacity and muscle fatigue biomarkers in ultra-marathoners.

### Study hypothesis

1. According to gut metagenomics, vegan ultra-marathoners have a more diverse microbiome than omnivorous ultra-marathoners.
2. Vegan ultra-marathoners have lower energy availability, macro- and micronutrient intakes than omnivore ultra-marathoners.
2. Ultra-marathoners following a long-term vegan diet have a greater antioxidant capacity than omnivorous ultra-marathoners.
3. There is no difference between vegan and omnivorous ultra-endurance athletes in terms of gut microbiome adaptation to ultra-marathon races.

### Trial design

This study is planned as a before and after study design. The participants will serve as their own control.

## Materials and methods

### Participants

Volunteer male and female ultra-marathoners will be recruited from the websites of the organizers of ultra-marathon events, vegan communities, runner magazines, and online running communities. In order for them to be eligible for the study, participants will need to take part in the Sri-Chinmoy ultra-marathon race in Basel, Switzerland. Potentially eligible participants will be identified by research staff. Ten vegan and ten omnivore participants will be randomly selected to enroll in the study using computer-generated random numbers. Randomization will be stratified by gender. Randomization and allocation will be performed by an independent researcher. The study will take place in Zurich and Basel, Switzerland.

#### Inclusion criteria

Participants will be classified as either vegan (no type of animal product consumption—dietary adherence <6 months) or omnivorous (consumption of any animal products—dietary adherence <6 months) based on self-reporting of diet consumed.

Principal inclusion criteria include:

1. For vegan athletes; athletes who do not consume any animal products for at least one year while active in running at least Marathon distances before the study:
2. For omnivorous athletes; athletes who consumed animal products for at least one year while active in running at least Marathon distances before the study
3. Ultra-marathoners with age between 18 to 49 years
4. Ultra-marathoners with adequate B12 levels (plasma B12 levels between 150–790 pmol / L and plasma homocysteine levels less than 15 μmol / L)
5. Ultra marathoners consuming enough energy intake (a CHO intake of more than 50% of their total calories)
6. Competing in the Sri-Chinmoy ultra-marathon race
7. No use of probiotics and antibiotics in the preceding 3 months
8. No history of acute or chronic illnesses.
9. No use of non-steroid anti-inflammatory drugs prior to and after the race

### Preliminary tests

Before enrollment, a 24-hour food record will be collected to determine adequate energy consumption. Ultra-endurance athletes consuming carbohydrates more than 50% of their total calories will be included to the study. In addition, plasma B12 and homocysteine levels will be assessed to define adequate B12 status. Athletes with plasma B12 levels between 150–790 pmol / L and plasma homocysteine levels less than 15 μmol / L will be considered to have adequate B12 levels.

Following full verbal and written explanation of the study procedures, participants will be asked to sign an informed consent form. After the enrollment of the study, research data will be collected from the participants at four steps (three visits to the laboratory and the race day) throughout the study. At the first visit (seven days before the race), participants will be invited to the exercise metabolism laboratory. Participants will be informed to come to the laboratory in a fastedstate. Fecal samples will be collected using fecal sample collection and preservative kit. Anthropometric measurements (body weight, height and fat-free mass) will be collected. An extended research questionnaire will be administered by research staff. Participants will be informed about how to keep detailed food and liquid diary and will be asked to keep dietary data throughout the study period (14 days). At the same time, a detailed activity log (activity, time and duration) will be kept for the participants.

The second visit will be performed within three to seven days before the race to measure maximum oxygen consumption using indirect calorimetry (Viasys Jager Master Screen CPX, PanGas AG, Dagmarsellen, Switzerland). Participants will be advised to follow their normal diet and to avoid strenuous exercise/ sports 24 hours before the VO_2_max test. Heart rate will be measured continuously by a heart rate monitor (Polar-S-810 heart rate monitor), and respiratory variables will be recorded every 30 s.

### Pre-race

Before starting the ultra-marathon race, body weight will be measured using a commercial scale. Blood samples will be drawn from an antecubital vein (two tubes/20 ml). The blood samples will kept on ice until it can be analysed.

### Post-race

Body weight will be measured and all food and liquid consumption during the race will be recorded by the dietician immediately after the race. Blood samples (two tubes / 20 ml) will be drawn at three points Post-Race: i) immediately after the race, ii) at 2 and iii) 24 hours after the end of the race. Participants will visit the laboratory to give blood samples 24 hours after the race (third visit).

Fecal samples will be collected from the participants’ at the forth visit. During this visit, all dietary data will be verified by the dietician.

### Description of measurements/methods/data collection

#### Research questionnaire

Participants will complete the extended research questionnaire which was applied in the NURMI study by Wirnitzer et al [31–34].

An extended research questionnaire form mainly includes questions related to health status, exercise/sports history (number of completed ultra-marathon competitions, year of first race, and number of training hours per week, etc.), supplement use, diet patterns before, during and after race. Additionally, the Physical Activity Readiness Questionnaire (PAR-Q) will be applied to determine current cardiovascular health [35]. Results of the questionnaires will be analyzed using SPSS statistic program version 23.0 (IBM, Armonk, NY).

#### Body composition assessment

To assess body composition of participants, they will be asked to visit the research laboratory in a fasted state (after an overnightfast) and refrain from caffeine (at least four hours), alcohol (at least two hours), cigarette (at least two hours), and not to exercise at high intensity for 24 hours before the visit.

Body weight and fat-free mass will be measured using a commercial scale Beurer BF 15 (Beurer GmbH, Ulm, Germany) with 0.1 kg accuracy. Height will be measured while participants are standing, head positioned in Frankfort horizontal plane, using a portable stadiometer (Seca 213, Hamburg, Germany). Bone mineral density and body fat percentage will be measured using dual- energy X-ray absorptiometry (DXA).

#### Energy availability

Individual RMR was estimated using the Mifflin-St. Jeor equation [36], as validated in ultra-endurance athletes previously [37].

All participants will be asked to keep a detailed activity logs (time, intensity, and duration) seven days before- and seven days after the race. Activity logs will provide the information on the intensity and duration of physical activity and training to calculate exercise energy expenditure (EEE) [38]. Corrected Metabolic Equivalent (MET) values from the Compendium of Physical Activities will be used to estimate the EEE recorded in the activity logs [39]. As previously stated by Guebels et al [40], only activities with an intensity greater than 4.0 METs will be included in the calculation. EEE is the sum of all exercises multiplied by the activity hours and FFM. EEE will be adjusted to remove the calories contributed by RMR for the duration of exercise [41] by subtracting RMR energy expenditure per hour of all activities reported at 4.0 METs or higher from the total EEE.

Participants will be informed about how to keep detailed food and liquid diary and will be asked to keep dietary data throughout the study period. Energy intake will be determined by the dietician using food and fluid records and analyzed with the Swiss Food Composition Database [42].

Energy availability will be calculated according to the formula below;

Energyintake−adjustedenergyexpenditure/fat-freemass(kg)

Recommended energy availability for athletes is defined as approximately 45 kcal.kg-1 and <30 kcal.kg-1 is defined as low energy availability [43].

*Maximum oxygen consumption measurement*. The maximum oxygen consumption (VO_2_max) will be measured using indirect calorimetry (Viasys Jager Master Screen CPX, PanGas AG, Dagmarsellen, Switzerland) by the method used in a previous study on male runners by Shing et. al [44]. The method includes an incremental running test performed on treadmill. The test protocol will start at 10 km h−1, 0% gradient with the speed increasing by 1 km h−1 each minute until a speed of 18 km h−1. After 1 min at 18 km h−1, the treadmill gradient will be increased by 1% each minute until volitional fatigue. Heart rate will be continuously measured Polar-S-810 heart rate monitor respiratory variables will be recorded every 30 s. Perceived exertion will be evaluated using Borg Scale 6 to 20 [45]. A higher score indicates a higher degree of perceived exhaustion.

#### Fecal sample collection

Fecal samples will be collected at two time points (seven days before and seven days after the race). The feces sample collection kit (Fe-Col^®^ Faecal Sample Collection Kits, UK) will be provided to the participants. They will be told about the feces collection protocol, described in detail by Wu et al. [46]. They will be required to bring the stool samples to the laboratory within the 45 minutes after collection. After stool samples will be delivered to the researchers, they will be first stored at -20°C for 24 hours, then will be delivered -80°C before the whole metagenomics analysis.

#### Blood parameters

Prior to study enrollment, plasma samples will be taken to analyze plasma vitamin B12 status and homocysteine levels to ensure all athletes have adequate vitamin B12 status. In addition, blood samples will be collected at four time points (immediately before- and immediately after the race, two and 24 hours after the end of the race) after enrolling the study. Plasma MDA, TAC (measured by FRAP assay), d-ROMs and HSP-70 will be analyzed to determine oxidant / antioxidant capacity. Serum ORM-1 will be analyzed to assess exercise-induced fatigue.

#### Environmental conditions

Environmental condition of the research laboratory will be standardized for all measurement (temperature 22–25 C). Furthermore, environmental and weather conditions of the race day will be recorded.

### Outcomes

#### Primary outcomes

1. Intestinal microbial adaptation according to applied diet evaluated by analysing faecal samples taken seven days before and seven days after the race using shotgun metagenomic analysis.

#### Secondary outcomes

1. Oxidative stress and muscle fatigue-related biomarkers measured using blood samples immediately before, at the end of 0 h, 2 h and 24 h of the race.

### Assessment of effectiveness and proof of evidence

#### Measurement of effectiveness

Effectiveness will be determined by the following methods and parameters:

- Sampling of blood (plasma MDA, d-ROMS, TAC, HSP-70, serum ORM-1)
- Measurement of lean body mass using DXA
- Measurement of body weight using a commercial scale (Bauer)
- Indirect calorimetry system, breath-by-breath method (Viasys Jager Master Screen CPX, PanGas AG, Dagmarsellen, Switzerland)
- Continuous heart rate monitoring while measuring VO_2_max (Polar-S-810 heart rate monitor)
- Rating of perceived exertion (Borg Scale)

#### Proof of evidence

Proof of evidence will be provided by the following parameters:

- VO_2_max
- Plasma MDA, d-ROMS concentration (Colorimetric/ fluorometric determination)
- Plasma HSP-70 concentration (ELISA/ in vitro quantitative measurement)
- Serum ORM-1 concentration (ELISA/ colorimetric determination)
- Gut microbiota structure (Shotgun metagenomic analysis)
- Bioinformatic interpretation (HumanN2 bioinformatic pipeline)

### Participant timeline

**Fig 1:**
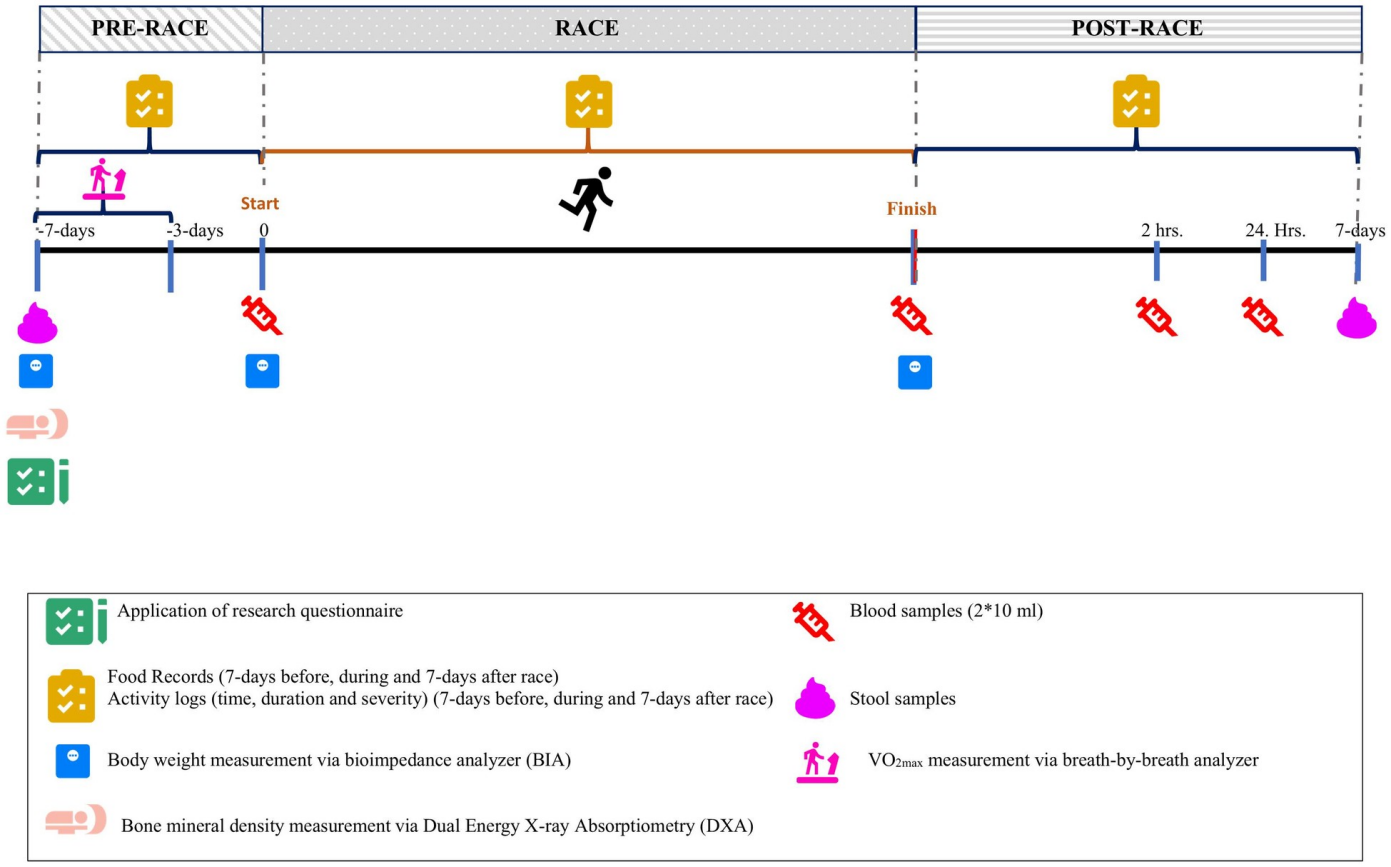
The flow chart of the research.

An overview of the research is presented in Fig 1.

### Sample size

The sample size (n = 20) provided 84% power to detect a difference with an effect size 0.7 at alpha 0.05 using two sided paired t-tests [47]. Considering the possible dropouts of the study or data clearance and laboratory testing processes, 35 participants from each dietary subgroup are planned to be included in the study.

### Statistical analysis

Statistical tests will be analyzed using SPSS version 23.0 software program (IBM, Armonk, NY) and R Software (Dusseldorf, Germany). Bioinformatic analysis will be performed using bioinformatics pipeline (HumanN2).

#### Fecal analysis

Fecal samples will be analyzed using next-generation sequencing technology (Illumina NextSeq500). Analysis and interpretation period is planned to perform at four steps; extraction of DNA from the samples, preparation of the sequence library, data analysis by sequencing, and interpretation of data using bioinformatics methods, respectively. In the first step, DNA will be extracted from fecal samples using the "Repeated bead beating plus column" method developed by Yu and Morrison [48]. In the second step, samples will be prepared using the modified Nextera XT DNA library preparation protocol (Illumina, California, USA) for Illumina NextSeq shotgun sequencing. DNA sequencing data obtained from FastQ files will first be quality checked to eliminate reading errors caused by human contamination using the NCBI Best Match Tagger (BMTagger).

Low-quality sequencing reads will be trimmed. Poor quality and duplicate reads will be removed using a combination of Picard and SAM tools [49, 50]. Taxonomic classifications of trimmed reads will be determined using Kraken [51] and Bracken [52] statistical methods. Functional profiling will be assessed using the Human Microbiome Project Unified Metabolic Analysis Network 2 (HUMAnN2; http://huttenhower.sph.harvard.edu/humann2) [53]. Taxonomic and functional analysis will be performed using R (Vienna, 2012) (version 3.5.1). Alpha and beta diversity will be calculated using the vegan package (version 2.5–2) in the R program [54]. A dendrogram will be created using the ape package (version 5.1) in the R program and visualized using iToL [55]. LEfSe, which allows the identification of pathways or taxa that characterize groups, will be used to identify species and pathways that characterize particular groups [56]. The significance threshold will be set as 0.05 and the LDA effect size threshold of 3 will be used for discriminative features. A heatmap map will be created using the R heatmap package to visualize the functional paths in HUMAnN2 [57].

### Quality control and quality assurance: Description of measures

#### Data security/disclosure of original documents

Data obtained from the study will be used exclusively for the study. All research staff will be required to protect research data to ensure anonymity and confidentiality of the participants. Only trained research staff will have access to the study at any given time. Original data such as questionnaire forms, research protocol and informed consent forms, and biological data will be stored in a locked and access controlled area. All electronic data will be stored on a password protected computer within a secure and access controlled area.

#### Duties on the part of the investigator

The research staff have confirmed that the study will be held on in accordance with the study protocol. Research staff will collect a 24-hour food record to check if all athletes are getting enough energy. Athletes consuming a CHO intake of more than 50% of their total calories will be included in the study. In addition, vitamin B12 status is a vital factor for the health status and performance capacity of ultra-endurance athletes, and the position paper on vegetarian diets suggested that vitamin B12 status should be closely monitored in vegan athletes. Therefore, the research staff will include ultra-endurance athletes with adequate B12 levels in the study by checking the plasma vitamin B12 and homocysteine status beforehand. The investigators will get permission to apply NURMI study questionnaire Step 2. The investigators has declared that all rights of participants will be respected, all materials related the study will be preserved, and all results will be reported accurately.

### Ethical principles

#### Ethics approval and consent to participate

Ethical approval was obtained from the Istanbul Medeniyet University Hospital Clinical Research Ethics Committee (Approval number 2013-KAEK-64). All participants will sign the informed consent form of the study prior to participation. The study will be conducted in accordance with the Declaration of Helsinki. Participants will be informed about the study including benefits and potential risks both verbally and in written form, and sign the informed consent form of the study prior to participation. Any protocol amendments, the informed consent, and all other forms of participant information related to the study and any other necessary documents will be reviewed by the Istanbul Medeniyet University Hospital Clinical Research Ethics Committee. In case of any amendments, subjects will receive a copy of any amendments to the written information provided to subjects.

#### Benefit for the participants

As a result of this study, participants will receive reliable information about their nutrition and performance status from health professionals. Individual results of the VO_2_max values, nutritional assessment according to food and fluid records, energy availability, body mineral density, and microbiota structure will be explained in detail to the participants. In case of any pathogenic variant is found, we will provide instructions to contact their healthcare provider to clarify the variant in a CLIA-certified laboratory. Further, they will receive individual consultations on obtaining adequate energy and nutrient intake by participating the research.

#### Description of risks

The increase of heart rate during incremental running test will be continuously monitored in order to prevent an adverse reaction, and only healthy participants with no history of cardiovascular disease will be invited to participate in the study. PAR-Q will be administred to assess current cardiovascular health [35]. Participants answering no to all questions will be considered as eligible for physical activity. The standard emergency equipment will be available in the research laboratory. Blood samples will be collected by a healthcare professional while the participants are positioned in a semi-reclined position to avoid risks associated with blood collection.

#### Trials status

Protocol version 1, June 24, 2020. The recruitment of participants has not started. The enrollment of the study is planning to start in January 2021, and is predicted to end May 2021.

## Discussion

Although the vegan diet has been widely preferable and studies claiming to be adequate for ultra-endurance athletes, there is still no comprehensive investigation of the impact of the vegan diet applied by ultra-endurance athletes. Therefore, we aim to provide an in-depth investigation of vegan diets by analyzing blood and fecal analysis in ultra-endurance athletes adhered to this diet for the long term. Previous studies considered vegan diets to be deficient for certain nutrients such as iron, calcium, zinc, iodine, omega-3 fatty acids, vitamin D, and vitamin B12 [58–60]. However, these nutrients may also be lacking in omnivores and vegetarians. For example, vitamin B12 has been found to be insufficient in older omnivores due to reduced absorption rate and some medications used [61]. Additionally, the common belief that omnivores consuming meat products do not have vitamin B12 deficiency may cause a vitamin B12 deficiency to go undiagnosed [62]. A well-planned vegan diet can provide all these nutrients for the athletic population [62, 63]. The Academy of Nutrition and Dietetics’ position statement on vegetarian diets has described well-planned vegan diets as healthy and nutritionally adequate diets that provide currently recommended dietary intakes and follow current dietary guidelines and are beneficial for preventing and treating certain diseases and are suitable for sedentary and athletes of all ages [64]. However, there is also a consensus statement that vegans need to carefully monitor their vitamin B12 intake and provide reliable vitamin B12 sources, including fortified foods or supplements as appropriate [61, 64]. Therefore, a carefully planned vegan diet with vitamin B12 supplement can meet all nutritional and energy needs of athletes [65, 66]. In case of vegan diets are found to be beneficial and sufficient, the study a level of evidence against the belief that the vegan diet may be insufficient for athletes and may provide the framework for future studies to assess veganism’s effects in athletic populations. Besides, the athletic population consumes meat products 2- and more fold compared to the other individuals, which creates a high risk for the world in terms of carbon footprint [67]. If a Vegan diet provides enough energy availability and balance macro- and micronutrients in the body, we can also recommend consuming the diet for the future of planet-saving. On the other hand, if vegan diets are found to be deficient or detrimental for either metabolism, including immunologic or oxidative factors or gut-host crosstalk, veganism’s adverse effects could be identified to inform athletes to eliminate its detrimental consequences.

By adding the measurements of oxidant/antioxidant- and muscle fatigue-related parameters, we seek to explore the impact of dietary patterns on body hormesis and fatigue levels in ultra-endurance athletes. Dietary intake data will analyze all diet composition, including nutrient and non-nutrient components, the main determinants of antioxidant defense. We may also determine whether the whole dietary pattern or food intake affects hormesis balance and endogenous antioxidant defense in athletes endured under extreme conditions.

One limitation of the study is its small sample size. We plan to include twenty ultra marathoners in the study, mainly due to the higher costs of the entire metagenomic analysis. However, our findings will provide pilot data for future studies investigating the gut metagenomics and endurance performance of ultra-endurance athletes.

In summary, we will scientifically evaluate the effect of vegan and omnivorous diets on gut metagenomics and the adaptation of the gut microbiome to extreme endurance exercise according to the vegan or omnivorous diet, which might provide a convenient means for regulation of nutritional requirements involving the basic and sports specific needs.

### Appendices

#### Plans for collection/laboratory evaluation, and storage of biological specimens

Stool samples will first have stored at -80 C before the shotgun metagenomics analysis. Blood samples will be collected 30 min-before and 0, 4, and 24 hours after race. Blood samples will be immediately centrifuged at 2000 rpm for 15 minutes after collection. Blood samples will be transferred to the cryotubes and stored at -80 C before further analysis.

## Supporting information

S1 File SPIRIT 2013 checklist: Recommended items to address in a clinical trial protocol and related documents*. (DOCX)

S2 File Trial protocol. (PDF)
