# Supplementary material for: Vegan vs. omnivore diets paradox: A whole-metagenomic approach for defining metabolic networks during the race in ultra-marathoners- a before and after study design
Source: PLoS One. 2021 Sep 23;16(9):e0255952. doi: 10.1371/journal.pone.0255952 (PMC8459986; doi:10.1371/journal.pone.0255952)
Supplement: S2 File — (PDF) [file pone.0255952.s002.pdf]

Trial Protocol

1. Research Name

Vegan vs. omnivore diets paradox: Determining the effects of dietary pattern on exercise-induced symptoms and a whole-metagenomic approach towards defining metabolic networks during the race in ultra-marathoners

2. Aims of the research

1. To investigate the effects of long-term vegan and omnivorous diet on gut metagenomics in ultra-marathoners,
2.To determine the adaptation of the gut microbiome to extreme endurance exercise according to the vegan or omnivorous diet in ultra-marathoners,
3. To determine energy availability, macro, and micronutrient intakes before, during, and after the race and compare the differences between vegan and omnivorous ultra-marathoners.
4. To explore the effects of long-term vegan and omnivorous diets on exercise capacity, oxidant/antioxidant capacity, and muscle fatigue in ultra-marathoners

3. Preliminary data supporting the research

With several elite athletes adopting veganism, a vegan diet has become more popular in the athletic population [1]. The vegan diet, which eliminates all animal products from an individual's diet, promotes health benefits that include a lower risk of cardiovascular disease, diabetes, obesity, hypertension, and cancer [2–4]. However, veganism has raised concerns about possible nutrient deficiencies, mainly protein, w-3, vitamin B12, zinc, and calcium [4,5]. Although the popularity of the vegan diet has increased among athletes, its effects on health and performance are still contentious. Additionally, there is a lack of research into the vegan dietary pattern in the athletic population [6].

According to a review by Pingitore et al. [7], while moderate exercise is defined as beneficial for endogenous antioxidant defenses, exhaustive/ long-duration performance could cause excessive release of stress hormones and products that alter the body redox balance. Therefore, although well-trained athletes might be resistant to increased reactive oxygen species (ROS), exhaustive exercise and inadequate recovery in the ultra-endurance athletes have been associated with the excessive production of lipid peroxidation and ROS, which alters body hormesis and causes both cellular and tissue damage [8,9]. The excessive release of stress factors causes an increase of lipopolysaccharides (LPS) translocation outside of the gut, resulting in exercise-induced endotoxemia, thus increasing permeability [10]. While lipid peroxidation and ROS can be determined in the formation of metabolic products such as malondialdehyde (MDA) [11], reactive oxygen metabolites (d-ROMs) [12], the body's endogenous antioxidant capacity can be evaluated by measuring plasma total antioxidant capacity (TAC) [13], a whole measurement of both enzymatic and non-enzymatic antioxidants. Previous studies have reported that consuming fruits and vegetables rich in antioxidants instead of taking antioxidant supplementation is suggested as the best approach to maintaining the body's oxidative balance [14–16]. A vegan diet, rich in both antioxidants and flavonoids, which are naturally occurring anti-inflammatory factors [1], could be an effective strategy to regulate the microbiome, reduce muscle fatigue and improve performance in ultra-endurance athletes.

Muscle fatigue is a common phenomenon caused by an alteration in metabolic conditions that restricts life and has detrimental effects on exercise performance [17].

Detection of muscle fatigue-related biomarkers, thus developing potential strategies to reduce muscle fatigue, is crucial for managing the pathologic conditions and enhancing muscle endurance in athletes, particularly during exhaustive exercise [18]. The occurrence of muscle fatigue depends on several multifactorial conditions, including energy metabolism biomarkers such as lactate, hydrogen ions, and oxidative stress and inflammatory biomarkers such as plasma ROS, TAC, and tumor necrose factor alpha (TNF-⍰) [19]. Additionally, immunologic biomarkers such as heat shock proteins (HSPs) [20] and orosomucoid (ORM) [21] are classified as a novel strategy to modulate muscle fatigue and immuno-modulating activity. Accordingly, Wan et al. [19] has claimed that foods rich in vitamins, minerals, flavonoids, and w-3 fatty acids are promising dietary factors that may reduce muscle fatigue in athletic populations. More research is needed to determine the effects of vegan and omnivorous diets on exercise-induced muscle fatigue and immunological conditions in athletes.

As long-term, low-grade energy and both macro- and micronutrient deficiencies are common in ultra-endurance athletes, they are at high risk for the occurrence of relative energy deficiency syndrome (RED-S) [22], including a decrease in bone mineral density and endocrine dysfunction, and reduced musculoskeletal function [23]. Previous literature has emphasized that vegans consume less energy and macro- and micronutrients than omnivores [3]. Whether vegan diets provide sufficient energy availability and preserve bone mineral density is a particular concern for vegan athletes, especially in ultra-endurance races.

Studies investigating the interaction between the gut microbiome and exercise performance in athletes have gradually increased over the past decades by searching the primary role of the gut microbiome on energy metabolism, immunomodulation, inflammatory response, and oxidative stress regulation [24]. Prior investigations have reported that exercise alone may contribute to alterations in the gut microbiome composition [25,26]. However, little is known about the interactions of microbial communities with exercise-related metabolic systems and communication pathways for exercise-related modification. Scheiman et al. [27] emphasized that microbiome modulation by detecting its performance-facilitating organism may be a critical exercise performance component. Metagenomic analyses allow direct comparison of the metabolism in the intestinal microbiota with the host's metabolic results and, integration of metabolomics and microbiome analysis could provide us to identify the microbial influence on host metabolism through bioactive metabolites [28–30]. Although a vegan diet appears to be beneficial for the gut microbiome by consuming foods that modulate/ regulate the microbiome [31], further work should focus on the efficacy of a vegan diet on host-microbiome interactions. To our knowledge, no study determines the vegan vs. omnivore diets' effects on gut metagenomics analyzed using a whole-metagenomic approach.

4. Research design

A controlled before and after study design

5. Selection of participants

Volunteer ultra-marathoners will be recruited from the websites of the organizers of ultra-marathon events, vegan communities, runner magazines, and online running communities. In order for them to be eligible for the study, participants will need to take part in the Sri-Chinmoy ultra-marathon race in Basel, Switzerland. Potentially eligible participants will be identified by research staff. Ten vegan and ten omnivore participants will be randomly selected to enroll in the study using computer-generated random numbers. Randomization

and allocation will be performed by an independent researcher. The study will take place in Zurich and Basel, Switzerland.

6. Inclusion Criteria

1) ultra-marathoners aged between 18 to 49 years
2) competing in the Sri-Chinmoy ultra-marathon race
3) no use of probiotics and antibiotics in the preceding 3 months
4) no history of acute or chronic illnesses.

7. Exclusion Criteria

1) ultra-marathoners aged < 18 or > 49 years
2) not competing in the Sri-Chinmoy ultra-marathon race
3) use of probiotics and antibiotics in the preceding 3 months
4) history of acute or chronic illnesses.

8. Primary Outcomes

1. Intestinal microbial adaptation according to applied diet evaluated by analysing faecal samples taken seven days before and seven days after the race using shotgun metagenomic analysis.

9. Secondary Outcomes

1. Oxidative stress and muscle fatigue-related biomarkers measured using blood samples immediately before, at the end of 0 h, 2 h and 24 h of the race.

10. Material and Methods

Preliminary Tests

Following full verbal and written explanation of the study procedures, participants will be asked to sign an informed consent form. After the enrollment of the study, research data will be collected from the participants at four steps (three visits to the laboratory and the race day) throughout the study. At the first visit (seven days before the race), participants will be invited to the exercise metabolism laboratory. Participants will be informed to come to the laboratory in a fasted state. Fecal samples will be collected using fecal sample collection and preservative kit. Anthropometric measurements (body weight, height and fat-free mass) will be collected. An extended research questionnaire will be administered by research staff. Triaxial accelerometry will be attached to the participants' waist, and will be secured with an elastic belt. The device will be worn continuously throughout the entire study period (seven days before and seven days after the race), except during activities that would submerge the accelerometer in water or if removed for legitimate reasons (e.g., discomfort during sleep). Participants will be informed about how to keep detailed food and liquid diary and will be asked to keep dietary data throughout the study period (14 days). At the same time, a detailed activity log (activity, time and duration) will be kept for the participants.

The second visit will be performed within three to seven days before the race to measure maximum oxygen consumption using indirect calorimetry (Viasys Jager Master Screen CPX, PanGas AG, Dagmarsellen, Switzerland). Participants will be advised to follow their normal diet and to avoid strenuous exercise/ sports 24 hours before the VO_2max test. Heart rate will be measured continuously by a heart rate monitor (Polar-S-810 heart rate monitor), and respiratory variables will be recorded every 30 s.

Pre-Race

Before starting the ultra-marathon race, body weight will be measured using a commercial scale. Blood samples will be drawn from an antecubital vein (two tubes/20 ml). The blood samples will kept on ice until it can be analyzed.

Post-Race

Body weight will be measured and all food and liquid consumption during the race will be recorded by the dietician immediately after the race. Blood samples (two tubes / 20 ml) will be drawn at three points Post-Race: i) immediately after the race, ii) at 2 and iii) 24 hours after the end of the race. Participants will visit the laboratory to give blood samples 24 hours after the race (third visit).

Fecal samples will be collected from the participants' at the forth visit. During this visit, all dietary data will be verified by the dietician.

10. 1. Data Collection Tools

Research Questionnaire

Participants will complete the extended research questionnaire which was applied in the NURMI study by Wirnitzer et al [32]. An extended research questionnaire form mainly includes questions related to health status, exercise/sports history (number of completed ultra-marathon competitions, year of first race, and number of training hours per week, etc.), diet patterns before, during and after race. Additionally, the Physical Activity Readiness Questionnaire (PAR-Q) will be applied to determine current cardiovascular health [33]. Results of the questionnaires will be analyzed using SPSS statistic program version 23.0 (IBM, Armonk, NY).

Body Composition Assessment

To assess body composition of participants, they will be asked to visit the research laboratory in a fasted state (after an overnightfast) and refrain from caffeine (at least four hours), alcohol (at least two hours), cigarette (at least two hours), and not to exercise at high intensity for 24 hours before the visit.

Body weight and fat-free mass will be measured using a commercial scale Beurer BF 15 (Beurer GmbH, Ulm, Germany) with 0.1 kg accuracy. Height will be measured while participants are standing, head positioned in Frankfort horizontal plane, using a portable stadiometer (Seca 213, Hamburg, Germany). Bone mineral density and body fat percentage will be measured using dual- energy X-ray absorptiometry (DXA).

Energy Availability

Individual RMR was estimated using the Mifflin-St. Jeor equation [34], as validated in ultra-endurance athletes previously [35].

All participants will be asked to keep a detailed activity logs (time, intensity, and duration) seven days before- and seven days after the race. Activity logs will provide the information on the intensity and duration of physical activity and training to calculate exercise energy expenditure (EEE) [36]. Corrected Metabolic Equivalent (MET) values from the Compendium of Physical Activities will be used to estimate the EEE recorded in the activity logs [37]. As previously stated by Guebels et al [38], only activities with an intensity greater

than 4.0 METs will be included in the calculation. EEE is the sum of all exercises multiplied by the activity hours and FFM. EEE will be adjusted to remove the calories contributed by RMR for the duration of exercise [39] by subtracting RMR energy expenditure per hour of all activities reported at 4.0 METs or higher from the total EEE.

Participants will be informed about how to keep detailed food and liquid diary and will be asked to keep dietary data throughout the study period. Energy intake will be determined by the dietician using food and fluid records and analyzed with the Swiss Food Composition Database [40].

Energy availability will be calculated according to the formula below;

Energy intake - adjusted energy expenditure/ fat-free mass (kg)

Recommended energy availability for athletes is defined as approximately 45 kcal.kg-1 and <30 kcal.kg-1 is defined as low energy availability [41].

Maximum Oxygen Consumption measurement

The maximum oxygen consumption (VO2max) will be measured using indirect calorimetry (Viasys Jager Master Screen CPX, PanGas AG, Dagmarsellen, Switzerland) by the method used in a previous study on male runners by Shing et. al [42]. The method includes an incremental running test performed on treadmill. The test protocol will start at 10 km h−1, 0% gradient with the speed increasing by 1 km h−1 each minute until a speed of 18 km h−1. After 1 min at 18 km h−1, the treadmill gradient will be increased by 1% each minute until volitional fatigue. Heart rate will be continuously measured Polar-S-810 heart rate monitor respiratory variables will be recorded every 30 s. Perceived exertion will be evaluated using Borg Scale 6 to 20 [43]. Higher score indicates higher exhaustion.

Fecal sample collection

Fecal samples will be collected at two time points (seven days before and seven days after the race). The feces sample collection kit (Fe-Col® Faecal Sample Collection Kits, UK) will be provided to the participants. They will be told about the feces collection protocol, described in detail by Wu et al. [44]. They will be required to bring the stool samples to the laboratory within the 45 minutes after collection. After stool samples will be delivered to the researchers, they will be first stored at -20 oC for 24 hours, then will be delivered -80 oC before the whole metagenomics analysis.

Blood parameters

Blood samples will be collected at four time points (immediately before- and immediately after the race, two and 24 hours after the end of the race). Plasma MDA, TAC (measured by FRAP assay), d-ROMs and HSP-70 will be analyzed to determine oxidant / antioxidant capacity. Serum ORM-1 will be analyzed to assess exercise-induced fatigue.

11. Statistical Analysis

Sample size

Statistical tests will be analyzed using SPSS version 23.0 software program (IBM, Armonk, NY) and R Software (Dusseldorf, Germany). Bioinformatic analysis will be performed using bioinformatics pipeline (HumanN2).

Fecal Analysis

Fecal samples will be analyzed using next-generation sequencing technology (Illumina NextSeq500). Analysis and interpretation period is planned to perform at four steps;

extraction of DNA from the samples, preparation of the sequence library, data analysis by sequencing, and interpretation of data using bioinformatics methods, respectively. In the first step, DNA will be extracted from fecal samples using the "Repeated bead beating plus column" method developed by Yu and Morrison [46]. In the second step, samples will be prepared using the modified Nextera XT DNA library preparation protocol (Illumina, California, USA) for Illumina NextSeq shotgun sequencing. DNA sequencing data obtained from FastQ files will first be quality checked to eliminate reading errors caused by human contamination using the NCBI Best Match Tagger (BMTagger).

Low-quality sequencing reads will be trimmed. Poor quality and duplicate reads will be removed using a combination of Picard and SAM tools [47,48]. Taxonomic classifications of trimmed reads will be determined using Kraken [49] and Bracken [50] statistical methods. Functional profiling will be assessed using the Human Microbiome Project Unified Metabolic Analysis Network 2 (HUMAnN2; http://huttenhower.sph.harvard.edu/humann2) [51]. Taxonomic and functional analysis will be performed using R (Vienna, 2012) (version 3.5.1). Alpha and beta diversity will be calculated using the vegan package (version 2.5-2) in the R program [52]. A dendrogram will be created using the ape package (version 5.1) in the R program and visualized using iToL [53]. LEfSe, which allows the identification of pathways or taxa that characterize groups, will be used to identify species and pathways that characterize particular groups [54]. The significance threshold will be set as 0.05 and the LDA effect size threshold of 3 will be used for discriminative features. A heatmap map will be created using the R heatmap package to visualize the functional paths in HUMAnN2 [55].

12. References

1. Rogerson D. Vegan diets: practical advice for athletes and exercisers. J Int Soc Sports Nutr. 2017;14: 36. doi:10.1186/s12970-017-0192-9
2. Craig WJ. Health effects of vegan diets. American Journal of Clinical Nutrition. 2009. doi:10.3945/ajcn.2009.26736N
3. Clarys P, Deliens T, Huybrechts I, Deriemaeker P, Vanaelst B, De Keyzer W, et al. Comparison of nutritional quality of the vegan, vegetarian, semi-vegetarian, pesco-vegetarian and omnivorous diet. Nutrients. 2014;6: 1318–1332. doi:10.3390/nu6031318
4. Appleby PN, Key TJ. The long-term health of vegetarians and vegans. Proceedings of the Nutrition Society. Cambridge University Press; 2016. pp. 287–293. doi:10.1017/S0029665115004334
5. Gilsing A, Crowe FL, Lloyd-Wright Z, Sanders T, Appleby PN, Allen NE, et al. Serum concentrations of vitamin B12 and folate in British male omnivores, vegetarians and vegans: results from a cross-sectional analysis of the EPIC-Oxford cohort study. Eur J Clin Nutr. 2010;64: 933–939. doi:10.1038/ejcn.2010.142
6. Barnard ND, Goldman DM, Loomis JF, Kahleova H, Levin SM, Neabore S, et al. Plant-based diets for cardiovascular safety and performance in endurance sports. Nutrients. MDPI AG; 2019. doi:10.3390/nu11010130
7. Pingitore A, Lima GPP, Mastorci F, Quinones A, Iervasi G, Vassalle C. Exercise and oxidative stress: Potential effects of antioxidant dietary strategies in sports. Nutrition. 2015;31: 916–922. doi:10.1016/j.nut.2015.02.005
8. Neubauer O, Reichhold S, Nics L, Hoelzl C, Valentini J, Stadlmayr B, et al. Antioxidant responses to an acute ultra-endurance exercise: impact on DNA stability and indications for an increased need for nutritive antioxidants in the early recovery phase. 2019 [cited 15 Mar 2019]. doi:10.1017/S0007114510001856

9. Knechtle B, Zingg MA, Rosemann T, Stiefel M, Rüst CA. What predicts performance in ultra-triathlon races? - a comparison between Ironman distance triathlon and ultra-triathlon. Open access J Sport Med. 2015;6: 149–59. doi:10.2147/OAJSM.S79273

10. Lamprecht M, Frauwallner A. Exercise, Intestinal Barrier Dysfunction and Probiotic Supplementation. Medicine and sport science. Karger Publishers; 2012. pp. 47–56. doi:10.1159/000342169

11. Nebl J, Drabert K, Haufe S, Wasserfurth P, Eigendorf J, Tegtbur U, et al. Exercise-Induced Oxidative Stress, Nitric Oxide and Plasma Amino Acid Profile in Recreational Runners with Vegetarian and Non-Vegetarian Dietary Patterns. Nutrients. 2019;11: 1875. doi:10.3390/nu11081875

12. Martarelli D, Verdenelli MC, Scuri S, Cocchioni M, Silvi S, Cecchini C, et al. Effect of a Probiotic Intake on Oxidant and Antioxidant Parameters in Plasma of Athletes During Intense Exercise Training. Curr Microbiol. 2011;62: 1689–1696. doi:10.1007/s00284-011-9915-3

13. Vezzoli A, Dellanoce C, Mrakic-Sposta S, Montorsi M, Moretti S, Tonini A, et al. Oxidative Stress Assessment in Response to Ultraendurance Exercise: Thiols Redox Status and ROS Production according to Duration of a Competitive Race. Oxid Med Cell Longev. 2016;2016: 1–13. doi:10.1155/2016/6439037

14. Owens DJ, Twist C, Cobley JN, Howatson G, Close GL. European Journal of Sport Science Exercise-induced muscle damage: What is it, what causes it and what are the nutritional solutions? Exercise-induced muscle damage: What is it, what causes it and what are the nutritional solutions? 2018 [cited 6 Mar 2019]. doi:10.1080/17461391.2018.1505957

15. Trapp D, Knez W, Sinclair W. Could a vegetarian diet reduce exercise-induced oxidative stress? A review of the literature. Journal of Sports Sciences. 2010. pp. 1261–1268. doi:10.1080/02640414.2010.507676

16. Yavari A, Javadi M, Mirmiran ; Parvin, Bahadoran Z. Exercise-Induced Oxidative Stress and Dietary Antioxidants. Asian J Sport Med. 2015;6: 24898. doi:10.5812/asjsm.24898

17. Wan J-J, Qin Z, Wang P-Y, Sun Y, Liu X. Muscle fatigue: general understanding and treatment. Exp Mol Med. 2017;49: 384. doi:10.1038/emm.2017.194

18. Knechtle B, Nikolaidis PT. Physiology and pathophysiology in Ultra-Marathon Running. Frontiers in Physiology. Frontiers Media SA; 2018. p. 634. doi:10.3389/fphys.2018.00634

19. Wan J-J, Qin Z, Wang P-Y, Sun Y, Liu X. Muscle fatigue: general understanding and treatment. Exp Mol Med. 2017; 384. doi:10.1038/emm.2017.194

20. Gomes Heck T, Schöler CM, Homem De Bittencourt PI. HSP70 expression: does it a novel fatigue signalling factor from immune system to the brain? Cell Biochem Funct. 2011;29: 215–226. doi:10.1002/cbf.1739

21. Lei H, Sun Y, Luo Z, Yourek G, Gui H, Yang Y, et al. Fatigue-induced Orosomucoid 1 Acts on C-C Chemokine Receptor Type 5 to Enhance Muscle Endurance. Sci Rep. 2016;6. doi:10.1038/srep18839

22. Torstveit MK, Fahrenholtz IL, Lichtenstein MB, Stenqvist TB, Melin AK. Exercise dependence, eating disorder symptoms and biomarkers of Relative Energy Deficiency in Sports (RED-S) among male endurance athletes. BMJ Open Sport Exerc Med. 2019;5: e000439. doi:10.1136/bmjsem-2018-000439

23. Hough PA, Earle J. Energy Balance During a Self-Sufficient, Multistage Ultramarathon. J Hum Perform Extrem Environ. 2017;13. doi:10.7771/2327-2937.1103

24. Mach N, Fuster-Botella D. Endurance exercise and gut microbiota: A review. J Sport Heal Sci. 2017;6: 179–197. doi:10.1016/J.JSHS.2016.05.001

25. Clark A, Mach N. Exercise-induced stress behavior, gut-microbiota-brain axis and diet: a systematic review for athletes. J Int Soc Sports Nutr. 2016;13: 43–64. doi:10.1186/s12970-016-0155-6
26. Zhao X, Zhang Z, Hu B, Huang W, Yuan C, Zou L. Response of Gut Microbiota to Metabolite Changes Induced by Endurance Exercise. Front Microbiol. 2018;9. doi:10.3389/fmicb.2018.00765
27. Scheiman J, Luber JM, Chavkin TA, MacDonald T, Tung A, Pham LD, et al. Meta-omics analysis of elite athletes identifies a performance-enhancing microbe that functions via lactate metabolism. Nature Medicine. Nature Publishing Group; 2019. pp. 1104–1109. doi:10.1038/s41591-019-0485-4
28. Tang ZZ, Chen G, Hong Q, Huang S, Smith HM, Shah RD, et al. Multi-omic analysis of the microbiome and metabolome in healthy subjects reveals microbiome-dependent relationships between diet and metabolites. Front Genet. 2019;10. doi:10.3389/fgene.2019.00454
29. MacCaferri S, Biagi E, Brigidi P. Metagenomics: Key to human gut microbiota. Digestive Diseases. 2011. pp. 525–530. doi:10.1159/000332966
30. Mangalam AK, Krementsov DN, Mondal RK, Kumar A, Malla MA, Dubey A, et al. Exploring the Human Microbiome: The Potential Future Role of Next-Generation Sequencing in Disease Diagnosis and Treatment. Front Immunol | www.frontiersin.org. 2019;1: 2868. doi:10.3389/fimmu.2018.02868
31. Tomova A, Bukovsky I, Rembert E, Yonas W, Alwarith J, Barnard ND, et al. The effects of vegetarian and vegan diets on gut microbiota. Front Nutr. 2019;6: 47. doi:10.3389/fnut.2019.00047
32. Wirnitzer K, Seyfart T, Leitzmann C, Keller M, Wirnitzer G, Lechleitner C, et al. Prevalence in running events and running performance of endurance runners following a vegetarian or vegan diet compared to non-vegetarian endurance runners: the NURMI Study. Springerplus. 2016;5: 1–7. doi:10.1186/s40064-016-2126-4
33. DM C, ML C, LL K, W D, N G. Physical activity readiness. B C Med J. 1975;17: 375–378.
34. Mifflin MD, St Jeor ST, Hill LA, Scott BJ, Daugherty SA, Koh YO. A new predictive equation for resting energy expenditure in healthy individuals. Am J Clin Nutr. 1990;51: 241–247. doi:10.1093/ajcn/51.2.241
35. Lanpir AD, Kocahan T, Deliceoğlu G, Tortu E, Bilgic P. Is there any predictive equation to determine resting metabolic rate in ultra-endurance athletes? Prog Nutr. 2019;21: 25–33. doi:10.23751/pn.v21i1.8052
36. Pereira MA, FitzerGerald SJ, Gregg EW, Joswiak ML, Ryan WJ, Suminski RR, et al. A collection of Physical Activity Questionnaires for health-related research. Med Sci Sports Exerc. 1997;29: S1-205. Available: https://experts.umn.edu/en/publications/a-collection-of-physical-activity-questionnaires-for-health-relat
37. Ainsworth BE, Haskell WL, Whitt MC, Irwin ML, Swartz AM, Strath SJ, et al. Compendium of physical activities: An update of activity codes and MET intensities. Med Sci Sports Exerc. 2000;32. doi:10.1097/00005768-200009001-00009
38. Guebels CP, Kam LC, Maddalozzo GF, Manore MM. Active women before/after an intervention designed to restore menstrual function: Resting metabolic rate and comparison of four methods to quantify energy expenditure and energy availability. Int J Sport Nutr Exerc Metab. 2014;24: 37–46. doi:10.1123/ijsnem.2012-0165

39. Heikura IA, Uusitalo ALT, Stellingwerff T, Bergland D, Mero AA, Burke LM. Low energy availability is difficult to assess but outcomes have large impact on bone injury rates in elite distance athletes. Int J Sport Nutr Exerc Metab. 2017.
40. Home - The Swiss Food Composition Database. [cited 23 Oct 2020]. Available: https://www.valeursnutritives.ch/en/
41. Loucks AB, Kiens B, Wright HH. Energy availability in athletes. J Sports Sci. 2011;29: S7–S15. doi:10.1080/02640414.2011.588958
42. Shing CM, Peake JM, Lim CL, Briskey D, Walsh NP, Fortes MB, et al. Effects of probiotics supplementation on gastrointestinal permeability, inflammation and exercise performance in the heat. Eur J Appl Physiol. 2014;114: 93–103. doi:10.1007/s00421-013-2748-y
43. Borg G. Psychophysical bases of perceived exertion. Med Sci Sports Exerc. 1982;14: 377–81. Available: http://www.ncbi.nlm.nih.gov/pubmed/7154893
44. Wu WK, Chen CC, Panyod S, Chen RA, Wu MS, Sheen LY, et al. Optimization of fecal sample processing for microbiome study — The journey from bathroom to bench. Journal of the Formosan Medical Association. Elsevier B.V.; 2019. pp. 545–555. doi:10.1016/j.jfma.2018.02.005
45. Nieman DC, Gillitt ND, Chen GY, Zhang Q, Sakaguchi CA, Stephan EH. Carbohydrate intake attenuates post-exercise plasma levels of cytochrome P450-generated oxylipins. PLoS One. 2019;14. doi:10.1371/journal.pone.0213676
46. Yu Z, Morrison M. Improved extraction of PCR-quality community DNA from digesta and fecal samples. Biotechniques. 2004;36: 808–812. Available: https://www.future-science.com/doi/pdf/10.2144/04365ST04
47. Li H, Handsaker B, Wysoker A, Fennell T, Ruan J, Homer N, et al. The Sequence Alignment/Map format and SAMtools. Bioinformatics. 2009;25: 2078–2079. doi:10.1093/bioinformatics/btp352
48. Picard Tools - By Broad Institute. [cited 21 Oct 2020]. Available: http://broadinstitute.github.io/picard/
49. Wood DE, Salzberg SL. Kraken: Ultrafast metagenomic sequence classification using exact alignments. Genome Biol. 2014;15: R46. doi:10.1186/gb-2014-15-3-r46
50. Lu J, Breitwieser FP, Thielen P, Salzberg SL. Bracken: Estimating species abundance in metagenomics data. PeerJ Comput Sci. 2017;2017: e104. doi:10.7717/peerj-cs.104
51. Franzosa EA, McIver LJ, Rahnavard G, Thompson LR, Schirmer M, Weingart G, et al. Species-level functional profiling of metagenomes and metatranscriptomes. Nat Methods. 2018;15: 962–968. doi:10.1038/s41592-018-0176-y
52. CRAN - Package vegan. [cited 21 Oct 2020]. Available: https://cran.r-project.org/web/packages/vegan/index.html
53. Letunic I, Bork P. Interactive tree of life (iTOL) v3: an online tool for the display and annotation of phylogenetic and other trees. Nucleic Acids Res. 2016;44: W242–W245. doi:10.1093/nar/gkw290
54. Segata N, Izard J, Waldron L, Gevers D, Miropolsky L, Garrett WS, et al. Metagenomic biomarker discovery and explanation. Genome Biol. 2011;12: R60. doi:10.1186/gb-2011-12-6-r60
55. heatmap function | R Documentation. [cited 21 Oct 2020]. Available: https://www.rdocumentation.org/packages/stats/versions/3.6.2/topics/heatmap

13. Originality of the research

Although the vegan diet has been widely preferable and studies claiming to be adequate for ultra-endurance athletes, there is still no comprehensive investigation of the impact of the vegan diet applied by ultra-endurance athletes. Therefore, we aim to provide an in-depth investigation of vegan diets by analyzing blood and fecal analysis in ultra-endurance athletes adhered to this diet for the long term. In case of vegan diets are found to be beneficial and sufficient, the study a level of evidence against the belief that the vegan diet may be insufficient for athletes and may provide the framework for future studies to assess veganism's effects in athletic populations. Besides, the athletic population consumes meat products 2- and more fold according to the other individuals. If a Vegan diet provides enough energy availability and balance macro- and micronutrients in the body, we can also recommend consuming the diet for the future of planet-saving. On the other hand, if vegan diets are detrimental for either metabolism, including immunologic or oxidative factors or gut-host crosstalk, veganism's adverse effects could be identified to inform athletes to inhibit its detrimental consequences.

By adding the measurements of oxidant/antioxidant- and muscle fatigue-related parameters, we seek to explore the impact of dietary patterns on body hormesis and fatigue levels in ultra-endurance athletes. Dietary intake data will analyze all diet composition, including nutrient and non-nutrient components, the main determinants of antioxidant defense. We may also determine whether the whole dietary pattern or food intake affects hormesis balance and endogenous antioxidant defense in athletes endured under extreme conditions.

14. Researchers' contribution

Conceptualization: Asli DEVRIM-LANPIR, Beat KNECHTLE
Formal analysis: Havvanur YOLDAS ILKTAC, Lee HILL
Investigation: Asli DEVRIM-LANPIR
Methodology: Asli DEVRIM-LANPIR, Beat KNECHTLE, Lee HILL
Project administration: Asli DEVRIM-LANPIR, Beat KNECHTLE, Thomas ROSEMANN
Supervision: Beat KNECHTLE

Lead Researcher (s): Beat KNECHTLE, Prof, MD
Asli DEVRIM LANPIR, PhD
